# Direct-Acting Antiviral Treatment for Acute Hepatitis C in Japanese Patients: Clinical Course and Outcomes

**DOI:** 10.7759/cureus.61724

**Published:** 2024-06-05

**Authors:** Hiroshi Okano, Katsumi Mukai, Akira Nishimura

**Affiliations:** 1 Gastroenterology, Suzuka General Hospital, Suzuka, JPN

**Keywords:** direct-acting antiviral treatment, people who inject drugs, sustained virologic response, chronicity, acute hepatitis c virus infection

## Abstract

We diagnosed six cases of acute hepatitis C virus (HCV) infection at our hospital between October 2003 and December 2022. During the same period, we diagnosed 402 cases of chronic HCV infection and 636 cases of acute hepatic injury. Acute HCV infection cases accounted for 1.4% of all HCV infections and 0.9% of all acute hepatic injury cases. The acute HCV infection group was younger, had more severe hepatitis, and exhibited higher levels of bilirubinemia compared to the chronic HCV infection group. Two acute HCV infection cases achieved spontaneous viral clearance, while the remaining four cases progressed to chronic infection and were treated with direct-acting antivirals (DAAs). Liver enzyme elevation and liver function deterioration did not differ significantly between the acute HCV and other acute liver injury groups. Notably, DAA treatment was equally effective for acute and chronic HCV cases (75% vs. 90%, p = 0.34).

Early DAA treatment in acute cases might contribute to interrupting viral transmission among high-risk populations, such as people who inject drugs or men who have sex with men. While there are currently no specific guidelines for acute HCV infection treatment in Japan, our findings suggest that DAA therapy should be initiated immediately following diagnosis. Further studies with larger patient cohorts are warranted to confirm these observations.

## Introduction

Now, almost all chronic hepatitis C virus (HCV) infection cases could have been cured by the treatment of direct-acting antivirals (DAAs) [1]. Sustained virologic response (SVR) resulting from HCV elimination brings significant benefits to the patients, such as lower all-cause mortality or lower incidence rates of hepatocellular carcinoma [2]. On the other hand, acute HCV infection is another clinical phenotype that is less common than chronic hepatitis in clinical practice, because symptomatic acute HCV infection occurs in only about 15% of patients who are infected with HCV [3]. HCV infection diagnosis in the acute phase is difficult since antibody production against HCV can be delayed [3]. Symptomatic patients with acute HCV infection with jaundice are more likely to eliminate HCV spontaneously than asymptomatic patients, but asymptomatic patients with acute HCV infection have lower spontaneous clearance of HCV, and early introduction of antiviral treatment is necessary for viral clearance [3,4]. In Japan, we do not have any suitable guidelines for acute HCV infection with clinical follow-up and timing of DAA treatment introduction so far, though there are already practice guidelines or treatment recommendations for acute HCV infection in the United States and Europe [5,6]. We have experienced six cases of acute HCV infection, including both definite cases and probable cases, in our hospital. In this report, we describe the characteristics and clinical course of these acute HCV hepatitis cases and analyze the comparison between the acute HCV infection group and the chronic HCV infection group. We also analyze the comparison between the acute HCV infection group and the other acute hepatitis group, especially hepatitis A, B, or E infection.

## Materials and methods

Patients and samples

This was an observational, single-center study conducted at Suzuka General Hospital. We retrospectively collected data from the medical records of patients between October 2003 and December 2022. During this period, we collected data on patients diagnosed with acute hepatic injury or chronic HCV infection. We included cases of viral hepatitis; drug-induced liver injury; autoimmune hepatitis with acute onset; ischemic hepatic injury; hypo-nutritional hepatic injury; and cryptogenic hepatic injury (unknown causes).

Oral informed consent, which included a statement of agreement for future scientific research using their samples, was obtained from each patient at their first medical examination in the Outpatient Department of Suzuka General Hospital [7]. Patients could also opt out of the study through a website. The present study was approved by the Ethics Committee of Suzuka General Hospital (Ethics Committee approved No.316).

HCV infection was diagnosed based on a patient being positive for both anti-HCV antibody (HCV-Ab) and HCV RNA or HCV RNA positive only. Serum HCV-Ab levels were measured by chemiluminescent enzyme immunoassay (CLEIA; Fujirebio Co., Ltd., Tokyo, Japan). Measurements of HCV RNA, HCV serogroup, and HCV genotype were performed at LSI Medience Corporation (Tokyo, Japan). Aspartate aminotransferase (AST), alanine aminotransferase (ALT), lactate dehydrogenase (LDH), alkaline phosphatase (ALP), g-glutamyltransferase (g-GT), and total bilirubin (T-Bil) levels were measured using the Cobas®8000 modular analyzer series (Roche Diagnosis K.K., Tokyo, Japan) or the LABOSPECT008 (Hitachi High-Tech Corporation, Tokyo, Japan). ALP levels were measured using the Japan Society of Clinical Chemistry (JSCC) method. Prothrombin time (PT) values were measured using the Sysmex CS-2500 automated coagulation analyzer (Sysmex Corporation). Blood specimens were obtained by venipuncture. The serum fraction obtained by centrifugation (2,100 x g at room temperature for 5 min) of the blood specimens was used for AST, ALT, LDH, ALP, g-GT, and T-Bil analyses. Plasma separated using a container with sodium citrate was used for the analysis of PT values. Statistical analysis was conducted using Bell Curve for Excel version 3.20 (Social Survey Research Information Co., Ltd.). Fisher’s exact test and the Mann-Whitney U test were used for comparisons between groups. One test was performed for each analysis.

## Results

Clinical characteristics of six acute HCV cases in a single center

Six cases (four males and two females) of acute HCV infection were identified in our hospital between October 2003 and December 2022 (Table 1 and Table 2).

**Table 1:** The six cases of acute hepatitis C infection occurred in our hospital. HCV Ab, anti-hepatitis C virus antibody; SVR, sustained virologic response; DAA, direct-acting antiviral. ^#1^Genotype 1 includes serotype 1. Genotype 2 includes serotype 2.

No.	Age	Sex	HCV Ab	HCV RNA	Transmission route	Diagnosis	^#1^Genotype	Clinical course
1	70	M	+	+	Suspected heterosexual intercourse	Probable	2	Spontaneous clearance
2	40	M	-	+	Unknown	Definite	2a	Spontaneous clearance
3	46	M	+	+	Suspected drug injection	Definite	2a	Chronicity and SVR after DAA treatment
4	33	F	+	+	Heterosexual intercourse	Definite	2a	Chronicity and SVR after DAA treatment
5	68	F	+	+	Unknown (iatrogenic?)	Definite	2b	Chronicity and SVR after DAA treatment
6	24	M	+	+	Suspected homosexual intercourse	Probable	1	Chronicity and DAA treatment

**Table 2:** Laboratory data on hepatitis onset in six cases of acute HCV infection. CBC, complete blood count; WBC, white blood cells; RBC, red blood cells; PT, prothrombin time; PT-INR, prothrombin time-international normalized ratio; AST, aspartate aminotransferase; ALT, alanine aminotransferase; LDH, lactate dehydrogenase; ALP, alkaline phosphatase; g-GT, g-glutamyltransferase; T-Bil, total bilirubin; HCVAb, anti-hepatitis C virus antibody; ND, no data; JSCC, Japan Society of Clinical Chemistry; IFCC, International Federation of Clinical Chemistry and Laboratory Medicine ^#1^: Serum ALP levels measured using the IFCC method could be calculated as 0.34 times the ALP levels measured using the JSCC method. ^#2^: Under anti-coagulation treatment.

	Case 1	Case 2	Case 3	Case 4	Case 5	Case 6	Reference range
CBC							
WBC (/mL)	6500	3700	6900	7300	3900	6300	3900-9800
RBC (x10^4^/mL)	540	613	544	408	336	539	427-570
Hemoglobin (g/dL)	17.0	18.5	16.7	10.7	10.7	14.8	13.5-17.6
Hematocrit (%)	50.5	49.1	48.0	31.5	31.9	44.8	39.8-51.8
Platelets (x10^4^/mL)	12.9	18.8	22.5	24.7	22.3	34.9	130-369
Coagulation							
PT (%)	74	77	^#2^40	100	101	113	70-130
PT-INR	1.17	1.15	^#2^1.67	1.00	1.00	0.94	0.91-1.14
Chemistry							
AST (IU/L)	572	874	621	314	959	209	10-35
ALT (IU/L)	1048	775	412	355	1078	683	10-35
LDH (IU/L) (IFCC)	340	844	494	177	751	207	124-222
ALP (IU/L) (IFCC)	^#1^251	^#1^157	^#1^109	^#1^122	^#1^177	208	72-113
g-GT (IU/L)	ND	538	584	55	61	231	8-60
T-Bil (mg/dL)	2.6	2.2	1.7	0.7	0.5	1.5	0.2-1.3
Viral markers							
HCV Ab (COI)	14.4(+)	0.1(-)	12.7(+)	7.6(+)	89.6(+)	61.9(+)	0.0-0.9
HCV-RNA (logIU/L)	2.0(+)	7.0(+)	7.6(+)	5.4(+)	6.7(+)	4.0(+)	0.0-1.1

The details of each case are as follows:

Case 1

A 70-year-old man with no prior history of hepatic disease was admitted to our hospital with acute hepatitis. He was found to be positive for both HCV-Ab and HCV RNA upon admission. No other viral infections, autoimmune liver diseases, medication history, or cardiopulmonary diseases were identified. After peaking at 1,176 IU/L, his ALT level spontaneously returned to the normal range. His T-Bil level also peaked at 5.7 mg/dL a few days after his ALT level peaked and then spontaneously returned to the normal range. The patient's HCV RNA level was 2.0 log IU/ml on admission and became negative after 60 days of spontaneous hepatitis resolution. He reported a history of sexual intercourse with a woman who had tattoos about one month before the onset of acute hepatitis. Based on this information, we diagnosed his acute hepatitis as probable acute HCV infection.

Case 2

A 40-year-old man was admitted to our hospital with acute hepatitis. He had no prior history of hepatic disease. Although initial laboratory tests showed negative HCV-Ab but positive HCV RNA, no other viral infections, autoimmune liver diseases, medication history, or cardiopulmonary diseases were identified. While he remained alert, his ALT level increased to 3,884 IU/L, and his prothrombin time-international normalized ratio (PT-INR) value worsened to 1.64 on day 4. Based on these findings, he was diagnosed with acute liver failure without hepatic encephalopathy due to acute HCV infection and underwent steroid pulse therapy with methylprednisolone 1g/day for three days. After steroid pulse therapy introduction, his hepatitis improved, and the PT-INR value also normalized. He seroconverted to HCV-Ab positivity after the hepatitis resolved. His HCV RNA viral load was 7.0 log IU/ml on admission and decreased after pulse therapy without the use of antiviral medication. Finally, HCV RNA became negative three months after hepatitis onset. We diagnosed the acute hepatitis as definite acute HCV hepatitis.

Case 3 and case 4

With regard to these two cases of acute HCV infection, we previously reported them in detail in the original case report [8]. These two cases were a married couple with acute HCV infection. Case 3 was a 46-year-old man, and case 4 was a 33-year-old woman. They presented to our hospital with elevated liver enzymes. The husband's laboratory results showed elevated liver enzymes (AST 621 IU/L, ALT 412 IU/L) and both HCV-Ab and HCV RNA positivity. Notably, he had been HCV-Ab negative two months prior. He had no prior history of hepatic disease and no history of recently started medication, autoimmune hepatic diseases, or other viral infections. Although he did have a history of cardiopulmonary disease, he did not experience any symptoms of exacerbation at the time of hepatitis onset. Given that HCV-Ab seroconversion was detected within the preceding six months of hepatitis, he was diagnosed with definite acute HCV infection. He reported being a drug injection user, suggesting that his HCV transmission likely occurred through this route. His viremia and elevated liver enzymes persisted for more than three months, indicating that his HCV infection did not achieve spontaneous clearance and had progressed. He did not develop jaundice during his clinical course. Therefore, his HCV infection was diagnosed as progressing to chronic hepatitis, and he was initiated on DAA treatment with sofosbuvir/ribavirin (SOF/RBV) for 12 weeks. He achieved SVR. On the other hand, the wife's laboratory data also showed positive HCV-Ab and HCV RNA with elevated liver enzymes (AST 314 IU/L and ALT 355 IU/L), although her HCV-Ab test had been negative 15 months prior. She had no prior history of liver disease and no evidence of other viral infections, autoimmune liver diseases, medication use, or cardiopulmonary disease. Although it was unknown whether her HCV-Ab was positive or negative six months before the development of hepatitis, genetic analysis confirmed transmission of the infection from her husband with acute HCV infection, as their HCV strains were identical. She also did not develop jaundice during her clinical course. Similarly, she was also diagnosed with acute HCV infection. Since her viremia also persisted for more than three months, indicating chronic infection, she received DAA treatment with 12 weeks of SOF/RBV and achieved SVR.

 Case 5

A 68-year-old woman was referred to our hospital with acute hepatitis. She had undergone surgery for colon carcinoma and started chemotherapy three months before the onset of hepatitis. Although they stopped the chemotherapy due to suspicion of drug-induced liver injury, her hepatitis continued. While her laboratory results at the time of surgery showed negative HCV-Ab, both HCV-Ab and HCV-RNA were detected at the onset of hepatitis. She had no prior history of liver disease and no evidence of other viral infections, autoimmune liver diseases, medication use other than chemotherapy, or cardiopulmonary disease. She was diagnosed with definite acute HCV infection. Though an iatrogenic transmission was suspected, the route of her HCV transmission could not be identified. Her ALT value increased to a peak of 1080 IU/L after a few days and then began to decrease, but it did not recover to the normal range, and her hepatitis continued. During her clinical course, she did not develop jaundice. Her HCV viremia became chronic, persisting for over three months after the onset of hepatitis. She was treated with eight weeks of DAA treatment with glecaprevir/pibrentasvir (GLE/PIB) for her HCV infection and achieved SVR.

 Case 6

A 24-year-old man was referred to our hospital with acute hepatitis. He had a skin rash and was diagnosed with syphilis by a dermatologist. Simultaneously, he was found to be positive for both HCV-Ab and HCV RNA. He reported occasional homosexual intercourse with men before the onset of both hepatitis and syphilis. He had no prior history of liver disease and no evidence of other viral infections, autoimmune liver diseases, medication use, or cardiopulmonary disease. His hepatitis was diagnosed as probable acute HCV infection due to the possibility of sexual transmission of both HCV and syphilis and his own history of risky sexual behavior. Although syphilis was cured by antibiotic treatment, his hepatitis persisted. During his clinical course, he did not develop jaundice. After six months of hepatitis onset, his HCV infection was diagnosed as chronic due to persistent HCV-RNA positivity and ongoing hepatitis. He was treated with DAAs using GLE/PIB for his HCV infection. He completed the eight-week DAA treatment, but he was lost to follow-up after the treatment ended.

Comparison of acute HCV infection cases to chronic HCV infection cases and other acute hepatitis cases

**Figure 1:**
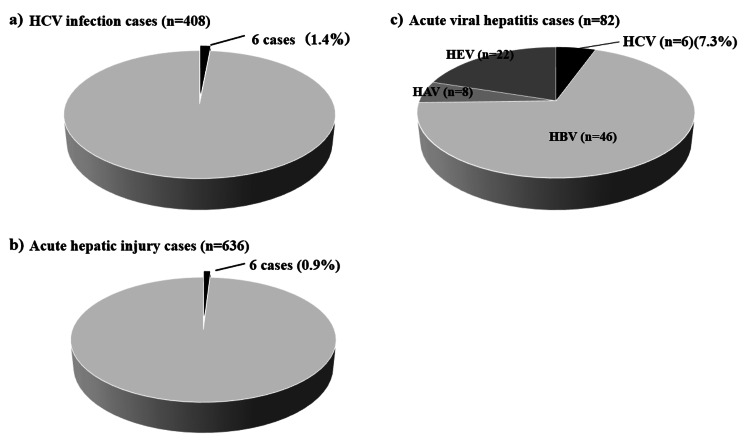
Incidence of hepatitis virus infections in our hospital (October 2003 - December 2022). Panel a): Acute HCV Infection vs. Chronic HCV Infection. This panel compares the number of cases diagnosed with acute hepatitis C virus infection (black) and chronic hepatitis C virus infection (gray) at our hospital between October 2003 and December 2022. Panel b): Acute HCV Infection vs. Other Acute Hepatic Injuries. This panel shows the incidence of acute hepatitis C virus infection (black) compared to other cases of acute hepatic injury (gray) diagnosed at our hospital between October 2003 and December 2022. Panel c): Acute Hepatitis Virus Infections (A, B, C, E). This panel depicts the incidence of acute infections with hepatitis viruses A, B, C, and E, at our hospital from October 2003 to December 2022. The black area specifically highlights the incidence of acute hepatitis C infection. HCV, hepatitis C virus

Between October 2003 and December 2022, our hospital observed 408 cases of HCV infection. Of these, six cases were acute HCV infection, representing 1.4% of all HCV infections (Figure 1a). In comparison, during the same period, the hospital observed 636 cases of acute hepatic injury (Figure 1b). Acute HCV cases accounted for 0.9% of all acute hepatic injury cases. Among these acute hepatic injury cases, 82 were viral hepatitis caused by hepatitis C, B, A, or E viruses (Figure 1c).

Hepatitis B virus infection was the most common cause of acute viral hepatitis, with HCV being the third most prevalent. Five out of the six acute HCV infection cases were genotype 2. In contrast, genotype 1 was the majority in chronic HCV infection (p=0.04). However, 90 cases of chronic HCV infection lacked genotype data (Table 3). Compared to the chronic HCV infection group, the acute HCV infection group was younger, had more severe hepatitis, and exhibited higher bilirubinemia levels (Table 4). However, there were no significant differences in hepatic function deterioration between the two groups (Table 4). We further compared the acute HCV infection group with the other acute hepatic injury group (Table 5). However, no significant differences were found in the data between these two groups. Similarly, no significant differences were observed between the acute HCV infection group and the other viral hepatitis groups (A, B, and E) (Table 6).

**Table 3:** The comparison of genotype distribution between acute hepatitis C infection and chronic hepatitis C infection. ND, no data. ^#1^Genotype 1 includes serotype 1, genotype 1a, and genotype 1b. Genotype 2 includes genotype 2, genotype 2a, and genotype 2b. ^#2^ p=0.04 (Fisher's exact test)

^#1^Genotype	Acute hepatitis C infection (n=6)	Chronic hepatitis C infection (n=402)
1	1^#2^	186^#2^
2	5^#2^	125^#2^
3	0	1
ND	0	90

**Table 4:** The comparison of acute hepatitis C infection and chronic hepatitis C infection. ALT, alanine aminotransferase; ALP, alkaline phosphatase; T-Bil, total bilirubin; PT, prothrombin time; JSCC, Japan Society of Clinical Chemistry; IFCC, International Federation of Clinical Chemistry and Laboratory Medicine. ^#1^: Serum ALP levels measured using the IFCC method could be calculated as 0.34 times the ALP levels measured using the JSCC method. ^#2^: The cases who were treated with anti-coagulation were excluded.

	Acute hepatitis C infection (n=6)	Chronic hepatitis C infection (n=402)	p-value
Age (year±SD)	43±18.7	66±15.6	0.012
Male/female	4/2	240/162	1.00
ALT (IU/L)	994.5±1285	48±85	<0.001
^#1^ALP (IU/L)	181±62	92±51	0.003
T-Bil (mg/dL)	1.9±5	0.8±0.9	0.007
^#2^PT	74±26	91±21	0.31

**Table 5:** The comparison of acute hepatitis C infection and the other acute hepatic injuries. ALT, alanine aminotransferase; ALP, alkaline phosphatase; T-Bil, total bilirubin; PT, prothrombin time; JSCC, Japan Society of Clinical Chemistry; IFCC, International Federation of Clinical Chemistry and Laboratory Medicine. ^#1^: Serum ALP levels measured using the IFCC method could be calculated as 0.34 times the ALP levels measured using the JSCC method. ^#2^: The cases who were treated with anti-coagulation were excluded.

	Acute hepatitis C infection (n=6)	The other acute hepatic injuries (n=630)	p-value
Age (year±SD)	43±18.7	58±20	0.12
Male/female	4/2	355/275	0.47
ALT (IU/L)	994.5±1285	485±1087	0.13
^#1^ALP (IU/L)	181±62	158±234	0.80
T-Bil (mg/dL)	1.9±5	1.3±5	0.49
^#2^PT	74±26	78±23	0.99

**Table 6:** The comparison of acute hepatitis C infection and acute hepatitis A, B and E infection. ALT, alanine aminotransferase; ALP, alkaline phosphatase; T-Bil, total bilirubin; PT, prothrombin time; JSCC, Japan Society of Clinical Chemistry; IFCC, International Federation of Clinical Chemistry and Laboratory Medicine. ^#1^: Serum ALP levels measured using the IFCC method could be calculated as 0.34 times the ALP levels measured using the JSCC method. ^#2^: The cases who are treated with anti-coagulation are excluded.

	Acute hepatitis C infection (n=6)	Acute hepatitis A, B and E infection (n=76)	p-value
Age (year±SD)	43±18.7	49.5±15.8	0.37
Male/female	4/2	66/10	0.21
ALT (IU/L)	994.5±1285	2175±1380	0.21
^#1^ALP (IU/L)	181±62	204±96	0.33
T-Bil (mg/dL)	1.9±5	6.15±5.8	0.14
^#2^PT	74±26	79±16	0.82

SVR at 24 weeks (SVR-24) in patients with acute and chronic HCV infection

**Figure 2:**
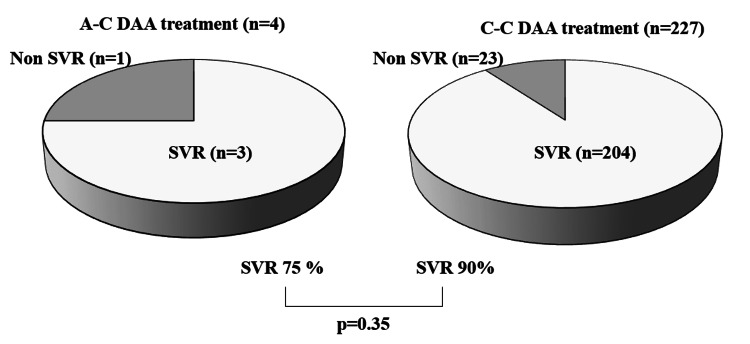
Treatment outcomes of direct-acting antivirals (DAAs) for acute and chronic HCV infection. This figure shows the results of DAA treatment for patients with acute hepatitis C virus infection (A-C DAA treatment) and chronic hepatitis C virus infection (C-C DAA treatment). Sustained virologic response (SVR): The SVR rate was 75% in the acute HCV infection group and 90% in the chronic HCV infection group. There was no statistically significant difference between the two groups. Non-SVR: This category includes both cases where HCV elimination was unsuccessful and cases where SVR at 24 weeks post-treatment (SVR-24) could not be determined due to loss to follow-up.

Out of four cases of acute HCV infection that progressed to chronic infection between October 2003 and December 2022, three achieved SVR-24 following treatment with DAAs. This corresponds to a 75% SVR-24 rate in the acute HCV infection group (Figure 2). One case with acute HCV infection did not achieve SVR-24, but the effect of DAA treatment could not be determined due to loss to follow-up. Conversely, among 227 chronic HCV infection cases during the same period, 204 achieved SVR-24, resulting in a rate of 90% (Figure 2). While the SVR-24 rate was lower in the acute HCV infection group compared to the chronic group, the difference was not statistically significant (p = 0.34) (Figure 2).

## Discussion

Among the six cases with acute HCV infection, two experienced symptomatic hepatitis with jaundice, resulting in spontaneous elimination of the virus without interferon or DAA treatment. Conversely, the remaining four cases with asymptomatic HCV hepatitis exhibited persistent viremia and progressed to chronic infection. These observations were consistent with previously reported findings [3,4]. In our cases, both patients who achieved spontaneous HCV clearance were infected with genotype 2 strains. Notably, three out of the four cases that developed chronic infection also harbored genotype 2. Hofer et al. reported that eight out of 12 cases with acute HCV infection spontaneously cleared the virus without antiviral therapy, including genotypes 1 and 3, but not genotype 2 [4]. Conversely, Hiura et al. documented a case of acute HCV genotype 1b infection progressing to chronicity under rituximab therapy for non-Hodgkin's lymphoma [9]. While factors like host ethnicity or immune status, including co-existing HIV infection, are known to influence spontaneous HCV elimination in acute infection [3], our data suggest that HCV genotype may not be a determining factor, as observed in our cases.

Diagnosing acute HCV infection can be challenging due to the lack of a definitive pathological test and confounding factors such as acute exacerbation of chronic HCV infection or other hepatitis conditions [3]. The gold standard for diagnosis involves documented seroconversion (antibody conversion from negative to positive) in a previously seronegative individual [3] or a positive HCV RNA test with a negative HCV-Ab test [10]. In our study, three out of six cases (cases 2, 3, and 5) met these criteria at their initial presentation of hepatitis. Cases 3 and 5 were documented as HCV-Ab negative before symptom onset and demonstrated seroconversion during their clinical course. One case was HCV-RNA positive and HCV-Ab negative at the onset, with seroconversion occurring after one month (case 2). The remaining two cases did not fulfill the aforementioned criteria (cases 1 and 6). However, we diagnosed them with acute HCV infection based on their reported risk behaviors prior to hepatitis onset and their lack of a previous history of liver disease. While we classified these cases as "probable" acute HCV infection, diagnosing similar cases in the future requires a more rigorous and meticulous approach.

**Figure 3:**
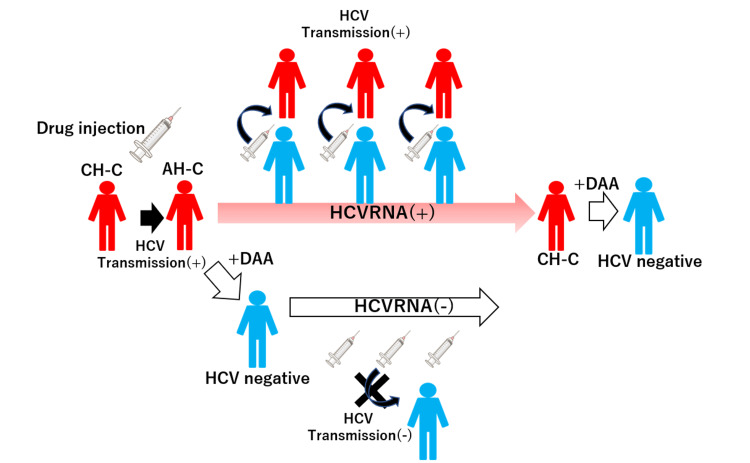
Impact of prompt DAA treatment for acute HCV infection in people who inject drugs (PWID) Pictograms: Red represents an HCV-positive individual, while blue represents an HCV-negative individual. Delayed DAA Treatment: Initiating DAA treatment only after HCV infection becomes chronic (red arrow pointing right) can allow for ongoing HCV transmission. Prompt DAA Treatment: Early DAA treatment upon diagnosis of acute HCV infection can disrupt the chain of HCV transmission (white arrows pointing right). DAA, direct-acting antiviral Image credit: Hiroshi Okano (Suzuka General Hospital), Katsumi Mukai (Suzuka General Hospital), and Akira Nishimura (Suzuka General Hospital).

Comparing the acute HCV infection group with chronic HCV infection groups or acute hepatic injury groups, acute HCV infection was relatively rare among these hepatic diseases in our hospital. The acute HCV infection group exhibited more severe hepatitis than the chronic HCV infection group. However, there were no significant differences in hepatic functional deterioration between the two groups. Similarly, the comparison between acute HCV infection and other hepatotropic virus infections did not show a significant difference. In our cases, only one case of acute HCV infection resulted in a spike in transaminase and PT-INR values, leading to hepatic failure during the clinical course. Based on our case and previous reports describing the rare phenomenon of acute hepatic failure caused by HCV [11], DAA treatment for acute HCV infection may be unnecessary until chronicity is established. On the other hand, cases 3 and 4 demonstrate the potential for transmission from an acutely infected individual via sexual intercourse. The case 3 patients acquired HCV through injection drug use (IDU), a population highly susceptible to acute HCV infection, accounting for up to 60% of newly acquired infections in developed countries [12]. Case 6, diagnosed with probable acute HCV infection, had a sexual history consistent with a known transmission route: homosexual intercourse. Men who have sex with men (MSM), especially those who are HIV-positive, are a major risk factor for HCV infection [12]. Particularly within the group of people who inject drugs (PWID), a single infected person can spread the virus to others through shared syringes. Rapid introduction of DAA treatment for acute HCV infection could disrupt this transmission chain within this group (Figure 3). Early DAA treatment for acute HCV infection could contribute to achieving the World Health Organization's (WHO) goal of HCV elimination by 2030. Since the introduction of blood donation screening and safer needle practices in healthcare, injection drug use has become the primary risk factor for HCV infection [13]. Therefore, we should prioritize DAA treatment for populations like PWID or MSM with acute HCV infection.

This study has limitations due to its small sample size and retrospective design. To gain a more comprehensive understanding of acute HCV infection and the efficacy of DAA treatment, future research should involve multicenter collaboration to analyze a larger pool of cases. The rarity of acute HCV infection in clinical practice necessitates such a large-scale approach. However, to our knowledge, no prior literature in Japan has analyzed the occurrence of acute HCV infection or the effectiveness of DAA treatment in a clinical setting. Therefore, we believe this article provides valuable insights into the diagnosis and DAA treatment of acute HCV infection in Japanese healthcare.

## Conclusions

We observed six cases of acute HCV infection. In most cases, the virus was eliminated spontaneously or through DAA treatment. In contrast to the management of chronic HCV infection, we propose early DAA treatment for acute HCV infection upon diagnosis. This approach is crucial to prevent further transmission, particularly among high-risk populations susceptible to HCV acquisition in Japan.
